# Association between binge eating and physical violence perpetration among U.S. college students

**DOI:** 10.1186/s40337-022-00700-z

**Published:** 2022-11-17

**Authors:** Kyle T. Ganson, Nicole E. Lisi, Julia O’Connor, Jason M. Nagata

**Affiliations:** 1 Factor-Inwentash Faculty of Social Work, University of Toronto, Toronto, Canada; 2 School of Social Work, University of Central Florida, Orlando, FL USA; 3 Department of Pediatrics, University of California, San Francisco, 550 16Th Street, 4th Floor, Box 0503, San Francisco, CA 94143 USA

**Keywords:** Binge eating, Physical violence perpetration, College, College students, Young adults

## Abstract

**Background:**

To date, no known research has explored the link between binge eating and physical violence perpetration despite overlapping psychological constructs that underpin these behaviors (i.e., emotion regulation difficulties, impulsivity). Therefore, this study aimed to determine the association between binge eating and self-reported physical violence perpetration.

**Methods:**

Cross-sectional data from four survey years (2016–2020) of the national (U.S.) Healthy Minds Study (*N* = 6210) were analyzed. Unadjusted (Independent samples *t* test) and adjusted (logistic regression) analyses were conducted to determine the associations between binge eating in the past four weeks and violence perpetration in the past 12 months, while adjusting for potential confounders.

**Results:**

The unadjusted mean number of days of binge eating was significantly higher among participants who reported physical violence perpetration (*M* = 2.6, SD = 5.2) compared to those who did not (*M* = 1.8, SD = 3.7). Logistic regression analysis demonstrated that each additional day of binge eating was associated with 5% higher odds (95% confidence interval 1.02–1.09) of self-reported physical violence perpetration, while adjusting for potential confounders.

**Conclusions:**

Results from this study are the first known to identify an association between binge eating and physical violence perpetration among U.S. college students. Findings are supported by the potential mechanistic overlap of emotion regulation and impulsivity associated with both binge eating and violence perpetration, underscoring the need for more research.

## Plain English summary

Both binge eating and physical violence perpetration are independently associated with impulsivity and emotion regulation difficulties, yet no known research has explored the link between these two behaviors. Findings from this study show that greater engagement in binge eating is associated with physical violence perpetration among U.S. college students, a particularly high-risk group for both eating disorder and physical violence involvement. These findings underscore the need for more research to understand the mechanisms that explain the link between these behaviors, as well as emphasize the need for prevention efforts on college campuses.

## Background

Adolescence and young adulthood (i.e., the college years) are particularly salient developmental phases when the codification of health and coping behaviors solidify [1, 2]. College students, in particular, are at elevated risk for mental health and interpersonal problems [3, 4]. Specifically, eating disorders are highly prevalent on college campuses, with upwards of 12% of college students at elevated risk for an eating disorder [5]. Disordered eating behaviors are also highly prevalent, with binge eating (40%) being more common than compensatory behaviors (e.g., purging behaviors; 30%) [5]. Binge eating episodes are characterized by consuming large amounts of food in relatively short time periods, lacking control over what and how much food is eaten, and often includes eating very quickly, eating regardless of hunger cues, and feelings of self-disgust, guilt, and depression [6]. The high risk of eating disorders and disordered eating behaviors is likely due to the increase in body dissatisfaction from early adolescence to young adulthood, with individuals reporting the highest body dissatisfaction during young adulthood [7]. The high occurrence of eating disorders and disordered eating behaviors is problematic given many associated adverse health and social effects [8–12].

Violence on college campuses is also relatively common. Among college students, 20% report dating violence victimization, including emotional, physical, and sexual violence, as well as stalking [13]. Relatedly, recent research has shown that nearly 5% of college students reported perpetrating violence in the past 12-months, with a significant increase over the last five years [14]. Violence involvement, including violence perpetration, like eating disorders and disordered eating behaviors, are associated with adverse outcomes [13, 15, 16]. Despite recent research documenting the association between physical violence perpetration and elevated eating disorder risk among college students [14], there is little knowledge of the specific eating disorder behaviors (e.g., binge eating) that are associated with physical violence perpetration. It is important to understand whether these forms of risk behaviors are connected to ensure proper assessment and intervention from on- and off-campus health professionals, public health professionals, as well as professionals within the justice system (i.e., police officers).

Contextualized by the adolescent and young adult developmental phase, research has documented the clustering of health risk behaviors, including disordered eating behaviors, violence, and substance use among young people [17–19]. The clustering of these behaviors may be explained by several factors. First, emotion dysregulation and the personality trait of impulsivity may be common factors that underlie these health risk behaviors [20, 21]. From this perspective, one may engage in disordered eating behaviors, violence, and/or substance use as mechanisms to manage unwanted negative emotions (i.e., maladaptive coping skills) [22]. Relatedly, adolescents and young adults may engage in these behaviors impulsively given neurological immaturity [23, 24], without thinking through potential consequences. Specifically, emotion regulation difficulty and impulsivity are common attributes of individuals with eating disorders, and it has been suggested that intense emotional states precede binge eating episodes. Thus, individuals likely engage in binge eating episodes in a maladaptive attempt to regulate negative emotions [25, 26]. Similar to binge eating, there is a strong correlation between aggression, the inability to regulate emotion, and impulsive behavior, with violence being understood as the inappropriate processing or expression of unpleasant emotions, and impulsivity as a high-risk factor for such conduct [27, 28].

Second, problem behavior theory posits that engagement in one problem behavior increases the likelihood of engagement in other problem behaviors [29, 30]. Through this theoretical lens, it may be that the clustering of health risk behaviors (i.e., binge eating and violence perpetration in this case), may occur given the unique risk and protective factors of the individual and the interaction between these individuals and the environment [29, 30]. This theory may also explain the high prevalence of substance use, including alcohol use and illicit drug use, among college students [31–34]. In fact, substance use may lead to further health risk behaviors (e.g., mental health problems, violence) [35]. Additionally, alcohol use and illicit drug use often coincide with engagement in, or are catalysts of, binge eating and violence perpetration [36–41].

This study aimed to test the hypothesis that binge eating and violence perpetration are associated given the clustering of health risk and problematic behaviors, as well as the underlying mechanisms of emotion regulation and impulsivity. Findings may guide future research on the clustering of problem behaviors among college students, as well as have implications for an interdisciplinary group of professionals.

## Methods

We analyzed pooled data from four survey years (2016–2017, 2017–2018, 2018–2019, 2019–2020) of the national (U.S.) Healthy Minds Study (HMS). HMS is an annual cross-sectional survey of the social and physical health and well-being of college students. Colleges and universities voluntarily elect to participate in HMS. At institutions with ≥ 4000 students, 4000 students were randomly invited to participate in the survey; at institutions with < 4000 students, all students were invited. To be eligible to participate, students must have been at least 18 years old and provided informed consent. Students were invited to participate via email and the survey was administered online via Qualtrics. Students were incentivized to participate using a prize drawing of Amazon gift cards. HMS was approved by the Health Sciences and Behavioral Sciences Institutional Review Board at the University of Michigan and all participating institutions.

### Measures

*Physical violence perpetration* was measured using the question: “Over the past 12 months, did you strike or physically injure anyone?” A dichotomous “yes” or “no” response was available. This measure has been used in prior research [14]. *Binge eating* was measured using the question, “Over the past 4 weeks (28 days), on how many days have you eaten an unusually large amount of food and have had a sense of loss of control at the time?” Response options ranged from zero (0) to 28 days. This measure is part of the widely used Eating Disorder Examination Questionnaire [42].

### Statistical analysis

HMS utilizes a module-based survey design, which limits the number of participants who received the binge eating measure each survey year. We excluded participants who did not respond to the binge eating and violence perpetration measures. This resulted in a sample of 6210 participants. We tested and found that the data was consistent with missing completely at random (Little’s MCAR test *p* > 0.05). Descriptive analyses were conducted to describe the sample characteristics. Unadjusted mean (M) number of days and standard deviations (SD) of binge eating in the past four weeks by self-reported physical violence perpetration in the past 12-months was estimated using Independent samples *t* test. Logistic regression analysis was conducted to estimate the association between number of days of binge eating in the past four weeks (independent variable) and self-reported physical violence perpetration in the past 12-months (dependent variable), while adjusting for age, race/ethnicity, gender, sexual orientation, body mass index (BMI; kg/m^2^), highest parental education, any illicit drug use (i.e., marijuana, cocaine, opioids, MDMA, etc.) in the past 30 days, and any alcohol use in the past two weeks. These variables were controlled for given prior research on related to binge-eating and violence perpetration [14, 36–41]. All analyses included preconstructed sample weighting to mitigate nonresponse bias and were conducted in 2021 using Stata 16.1.

## Results

**Fig. 1:**
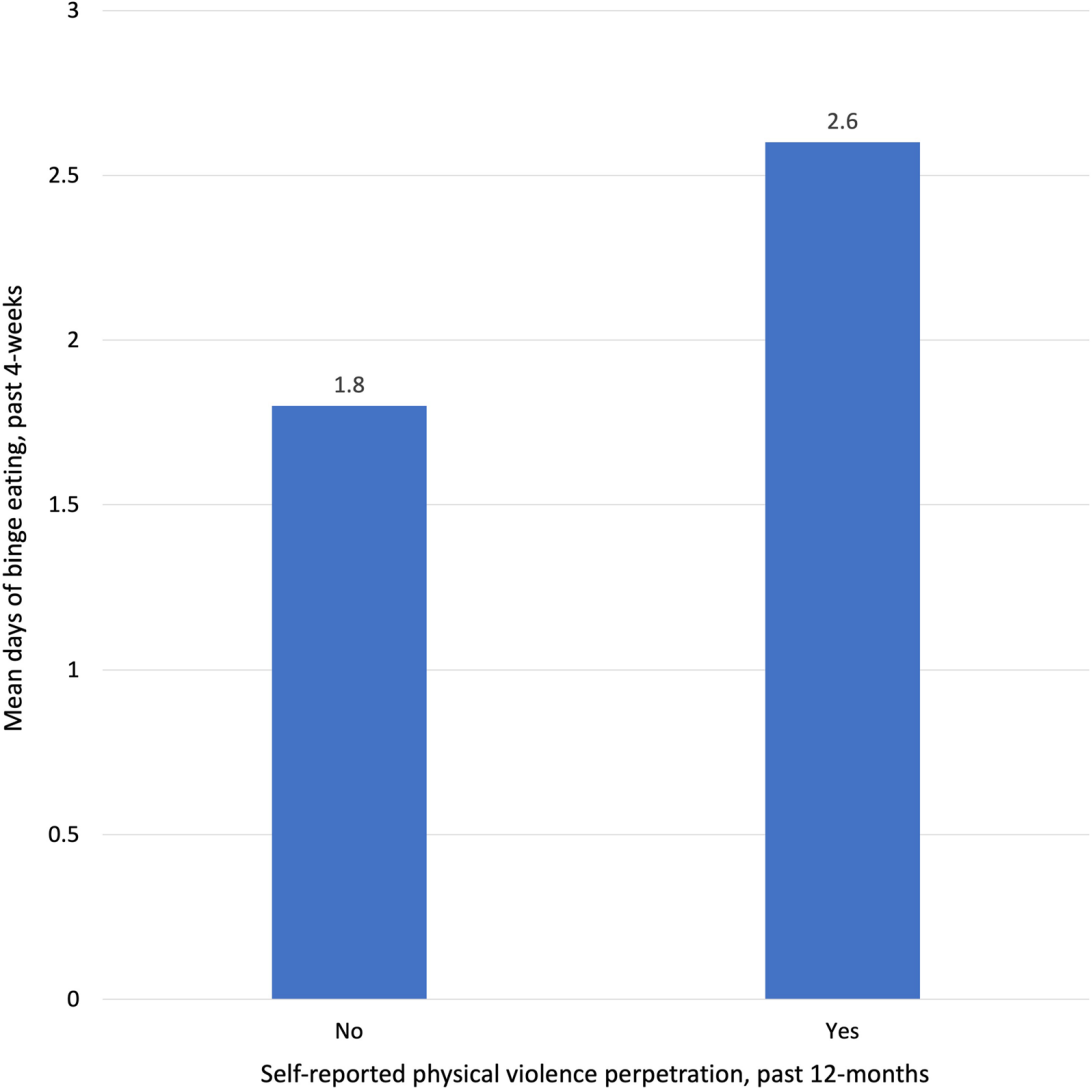
Unadjusted mean days of binge eating in the past four weeks by self-reported physical violence perpetration in the past 12-months among participants in the 2016–2020 Healthy Minds Study (*N* = 6210). Mean days of binge eating was significantly higher (*p* < 0.001) among those who reported physical violence perpetration.

Among the diverse sample of 6,210 college student participants, the mean number of days of binge eating in the past four weeks was 1.8 (SD = 3.7) and 3.5% reported physical violence perpetration in the past 12-months (Table 1). The unadjusted mean number of days of binge eating was higher among participants who reported physical violence perpetration in the past 12-months (*M* = 2.6, SD = 5.2, *p* < 0.001; Fig. 1) compared to those who did not (*M* = 1.8, SD = 3.7). Logistic regression analysis demonstrated that each additional day of binge eating in the past four weeks was associated with 5% higher odds of self-reported physical violence perpetration in the past 12-months (95% confidence interval 1.02–1.09, *p* = 0.001), while adjusting for potential confounders. In a posthoc analysis, we found no evidence of effect modification by gender on the association between binge eating and physical violence perpetration.

**Table 1:** Demographic and sample characteristics of participants from the 2016–2020 Healthy Minds Study (*N* = 6210)

	Mean (SD)/%
Age	22.3 (5.8)
Body mass index (kg/m^2^)	24.2 (4.8)
Race/ethnicity	
White or Caucasian, non-Hispanic, non-Arab	64.4%
Black or African American, non-Hispanic	5.0%
Hispanic/Latino/a	4.9%
Asian or Asian American	13.1%
American Indian, Alaskan Native, Native Hawaiian or Pacific Islander	0.2%
Arab/Middle Eastern or Arab American	1.2%
Other race/ethnicity	0.9%
More than 1 race/ethnicity	10.3%
Gender	
Men	42.1%
Women	57.9%
Sexual orientation	
Heterosexual	81.3%
Gay or lesbian	5.7%
Bisexual	8.0%
Queer, questioning, or other	4.9%
Highest parental education	
High school or less	8.0%
Some college or more	92.0%
Physical violence perpetration, past 12-months	3.5%
Days binge eating, past four weeks	1.8 (3.7)

Preconstructed nonresponse sample weighting was applied to all analyses

*SD* standard deviation

## Discussion

This study aimed to determine the association between binge eating and physical violence perpetration among a national sample of college student participants. Results showed significant associations in both unadjusted and adjusted analyses. Among over 6200 college student participants, the average number of days of binge eating at the time of the study was higher among participants who reported physical violence perpetration, and each additional day of binge eating was associated with 5% higher odds of physical violence perpetration. This finding aligns with and expands prior research showing a significant association between elevated eating disorder risk and physical violence perpetration among college students [14], as well as eating disorders and aggressive and impulsive behaviors [43, 44].

Contextualized by the clustering of health risk and problem behaviors, the association between binge eating and physical violence perpetration may be explained by the overlapping psychological constructs of difficulty with emotion regulation and impulse control [25–28]. Importantly, in the adjusted analysis, the association between binge eating and physical violence perpetration was independent of any alcohol use and any illicit drug use. This is important considering that substance use can also coincide with disordered eating behaviors and violence involvement [17–19].

The findings from this study have important implications for future research, as well as an interdisciplinary group of professionals. Future research is needed to determine the prospective relationship between binge eating and physical violence perpetration and empirically identify the mechanisms underlying these behaviors. This may include the use of a prospective cohort study to assess the relationship between binge eating and physical violence perpetration over time, as well as qualitative analyses that investigate the lived experiences of young people who engage in both binge eating and physical violence perpetration. The use of specific emotion regulation (e.g., Difficulties in Emotion Regulation Scale) [45] and impulsivity (e.g., Barratt Impulsiveness Scale) [46] measures should also be included in future research to assess whether these constructs empirically mediate the relationship between binge eating and physical violence perpetration. Healthcare professionals should understand that health risks and problem behaviors may cluster together, and consider screening for both binge eating and physical violence perpetration when one or both are present. Utilizing targeted interventions aimed at increasing emotion regulation and impulse control capabilities with clients may be warranted to prevent or reduce both binge eating and physical violence perpetration [47]. Additionally, public health programs and institutions of higher education should consider the overlapping nature of mental health problems and interpersonal violence to inform and tailor student health and mental health supports (e.g., information regarding healthy living behaviors and coping, support for eating disorders) and violence prevention efforts (e.g., de-escalation techniques, education on healthy relationships). Finally, it may be particularly important to provide campus police sufficient education and training on the overlap between mental health problems (i.e., eating disorder behaviors such as binge eating) and violence among college students. This may generate new avenues for assessment and treatment of eating disorders and violence.

There are several strengths and limitations of this study to note. Strengths of this study include the use of a large, diverse, and national sample of college students. Limitations include the cross-sectional survey design, which limits causal inference; however, this study did not specifically aim to identify the directionality between binge eating and violence perpetration, as it is likely that the relationship is bidirectional given the clustering of health risk and problem behaviors. Additionally, the measures are based on self-report, which may increase social desirability bias, and there is the potential for unmeasured confounders that may influence the relationship.

## Conclusion

The results from this study show that binge eating and physical violence perpetration are associated within a national sample of college student participants. This association may be explained by the clustering of health risk and problem behaviors, as well as the psychological constructs of emotion regulation and impulse control difficulty common among individuals who engage in these behaviors. Future research is needed to empirically identify these underlying mechanisms.

## Data Availability

The Healthy Minds Study is available to researchers. Visit https://healthymindsnetwork.org/ for more information.
